# Mechanistic details of the actinobacterial lyase-catalyzed degradation reaction of 2-hydroxyisobutyryl-CoA

**DOI:** 10.1016/j.jbc.2021.101522

**Published:** 2021-12-22

**Authors:** Michael Zahn, Gerhard König, Huy Viet Cuong Pham, Barbara Seroka, Ryszard Lazny, Guangli Yang, Ouathek Ouerfelli, Zenon Lotowski, Thore Rohwerder

**Affiliations:** 1 Centre for Enzyme Innovation, School of Biological Sciences, Institute of Biological and Biomedical Sciences, University of Portsmouth, Portsmouth, United Kingdom; 2 Department of Environmental Microbiology, Helmholtz Centre for Environmental Research - UFZ, Leipzig, Germany; 3 Faculty of Chemistry, University of Bialystok, Bialystok, Poland; 4 Organic Synthesis Core Facility, Memorial Sloan Kettering Cancer Center (MSKCC), New York, New York, USA

**Keywords:** x-ray diffraction, Actinomycetospora, 2-hydroxyacyl-CoA synthase, mono-carbon extension, glycolyl-CoA, carbonyl compounds, formate assimilation, oxalyl-CoA decarboxylase, 2-HIB-CoA, 2-hydroxyisobutyryl-CoA, 2-HIBA, 2-hydroxyisobutyric acid, AcHACL, 2-hydroxyacyl-CoA lyase from *Actinomycetospora chiangmaiensis*, ALS, acetolactate synthase, BsALS, acetolactate synthase from *Bacillus subtilis*, dzThDP, 3-deazathiamine diphosphate, HACL, 2-hydroxyacyl-CoA lyase, HsHACL1, human 2-hydroxyacyl-CoA lyase 1, HsHACL2, human 2-hydroxyacyl-CoA lyase 2, MeOXC, oxalyl-CoA decarboxylase from *Methylorubrum extorquens*, OfOXC, oxalyl-CoA decarboxylase from *Oxalobacter formigenes*, OXC, oxalyl-CoA decarboxylase, RuHACL, 2-hydroxyacyl-CoA lyase from *Rhodospirillales* bacterium URHD0017, ThDP, thiamine diphosphate, TzDP, thiazolone diphosphate

## Abstract

Actinobacterial 2-hydroxyacyl-CoA lyase reversibly catalyzes the thiamine diphosphate-dependent cleavage of 2-hydroxyisobutyryl-CoA to formyl-CoA and acetone. This enzyme has great potential for use in synthetic one-carbon assimilation pathways for sustainable production of chemicals, but lacks details of substrate binding and reaction mechanism for biochemical reengineering. We determined crystal structures of the tetrameric enzyme in the closed conformation with bound substrate, covalent postcleavage intermediate, and products, shedding light on active site architecture and substrate interactions. Together with molecular dynamics simulations of the covalent precleavage complex, the complete catalytic cycle is structurally portrayed, revealing a proton transfer from the substrate acyl Cβ hydroxyl to residue E493 that returns it subsequently to the postcleavage Cα-carbanion intermediate. Kinetic parameters obtained for mutants E493A, E493Q, and E493K confirm the catalytic role of E493 in the WT enzyme. However, the 10- and 50-fold reduction in lyase activity in the E493A and E493Q mutants, respectively, compared with WT suggests that water molecules may contribute to proton transfer. The putative catalytic glutamate is located on a short α-helix close to the active site. This structural feature appears to be conserved in related lyases, such as human 2-hydroxyacyl-CoA lyase 2. Interestingly, a unique feature of the actinobacterial 2-hydroxyacyl-CoA lyase is a large C-terminal lid domain that, together with active site residues L127 and I492, restricts substrate size to ≤C5 2-hydroxyacyl residues. These details about the catalytic mechanism and determinants of substrate specificity pave the ground for designing tailored catalysts for acyloin condensations for one-carbon and short-chain substrates in biotechnological applications.

2-Hydroxyacyl-CoA lyase (HACL) enzymes belong to the thiamine diphosphate (ThDP)-dependent decarboxylase superfamily (1) and catalyze the reversible cleavage of 2-hydroxyacyl-CoA thioesters to the corresponding carbonyl compound and formyl-CoA (Fig. 1). The first HACL (HsHACL1) has been described in the human peroxisomal α-oxidation pathway (2) where it is involved in the decomposition of 3-methyl-branched fatty acids, *e.g.*, by cleaving 2-hydroxyphytanoyl-CoA (C20 acyl residue). Mechanistically related to the HACL reaction is the ThDP-dependent decarboxylation of oxalyl-CoA to carbon dioxide and formyl-CoA (Fig. 1) catalyzed by bacterial oxalyl-CoA decarboxylase (OXC) (3). Until recently, the human HACL homologs (HsHACL1 and HsHACL2) and bacterial OXC were the only known ThDP-dependent enzymes characterized for the formyl-CoA removal from acyl-CoA substrates (2, 4). In 2019, however, Chou *et al.* (5) reported the cleavage of 2-hydroxyoctadecanoyl-CoA into heptadecanal and formyl-CoA by an HACL from *Rhodospirillales* bacterium URHD0017 (RuHACL). Interestingly, the latter enzyme can also catalyze the condensation of formyl-CoA with various short-chain aldehydes and ketones. The employment of HACL enzymes as synthases to produce the acyloin 2-hydroxyacyl-CoA has recently attracted attention as a new tool for mono-carbon extension reactions (6, 7). However, the reengineering of HACL to improve efficiency toward target substrates is impeded by the lack of structural data. Consequently, specific details on substrate binding as well as on the reaction mechanism are missing.

**Figure 1:**
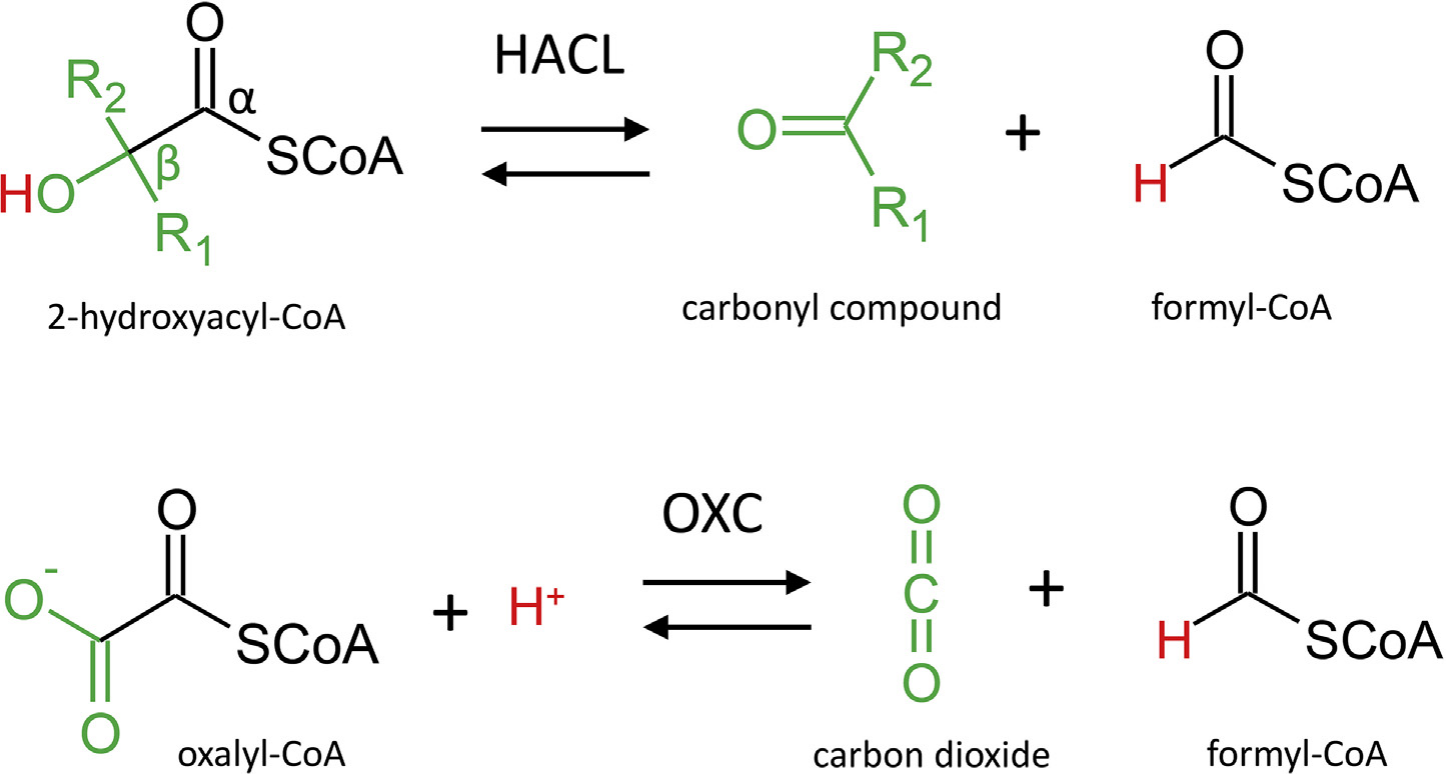
**Schematic comparison of HACL and OXC-catalyzed C-C cleavage reaction**.

Thus far, only one HACL structure showing substrate-free RuHACL in an open conformation with the bound ThDP analog thiamine thiazolone diphosphate (TzDP) has been deposited (PDB ID: 6XN8). In contrast, OXC from *Oxalobacter formigenes* (OfOXC) is well studied (8, 9), and structures with noncovalently bound oxalyl-CoA and formyl-CoA as well as with the covalent postdecarboxylation intermediate have been analyzed. Since both OXC and HACL share common features, such as the binding of an acyl-CoA substrate and the release of formyl-CoA (Fig. 1), it is tempting to transfer the HACL reaction to an OXC template. Accordingly, a quadruple mutant of the OXC from *Methylorubrum extorquens* (MeOXC) has recently been established for the condensation of formyl-CoA with formaldehyde by directed evolution (10). However, the analogy between OXC and HACL is limited. Accommodation, *e.g.*, of the negatively charged carboxyl group of oxalyl-CoA may require interactions with active site amino acids not compatible with the binding of the uncharged 2-hydroxyacyl residue. Presumably more important, the HACL reaction proceeds *via* deprotonation of the Cβ hydroxyl group during Cα-Cβ cleavage (Fig. 1), requiring an appropriate proton acceptor within the active site. Likely, these differences in substrate specificity and reaction mechanism are reflected in structural peculiarities in HACL that await elucidation.

In the Gram-negative bacterium *Aquincola tertiaricarbonis* L108, the short-chain and tertiary branched carboxylic acid 2-hydroxyisobutyric acid (2-HIBA) is formed during the degradation of the xenobiotic fuel oxygenate methyl *tert*-butyl ether (11). We have recently characterized and solved the crystal structures of two enzymes involved in that pathway (12, 13). The enzyme 2-HIBA-CoA ligase activates 2-HIBA with CoA to form 2-HIB-CoA. In a subsequent reaction, 2-HIB-CoA mutase forms the isomer (*S*)-3-hydroxybutyryl-CoA. Recently, 2-HIBA was found to be a marker for several human diseases, such as obesity and diabetes mellitus (14, 15). In addition, lysine 2-hydroxyisobutyrylation is a newly discovered posttranslational modification (16, 17), and the responsible acyl transferases have been identified (18). However, the underlying pathway of the *in vivo* synthesis of 2-HIBA and 2-HIB-CoA still remains elusive. In this context, a novel 2-HIBA degradation pathway was recently discovered in the actinobacterial strain *Actinomycetospora chiangmaiensis* DSM 45062, which contains an enzyme homolog for 2-HIBA-CoA ligase but no homolog for 2-HIB-CoA mutase (19). Instead, a HACL enzyme (AcHACL) cleaves 2-HIB-CoA into acetone and formyl-CoA. Moreover, 2-butanone is formed from 2-hydroxy-2-methylbutyryl-CoA at comparable rates (19). In contrast to HsHACL1, HsHACL2, and RuHACL, which preferentially use long-chain substrates that are not relevant in biotechnology, AcHACL’s native substrate for the synthase reaction is the relatively short acetone, thereby offering great potential for the design of formate assimilation reactions.

Here, we report the first crystal structure of a HACL enzyme in complex with the inactive cofactor 3-deazathiamine diphosphate (dzThDP) and substrate 2-HIB-CoA. Moreover, AcHACL was cocrystallized with ThDP and substrate 2-HIB-CoA to capture the covalent postcleavage intermediate as well as the free reaction products acetone and formyl-CoA. Modeling of the corresponding covalent precleavage intermediate revealed an important interaction with residue E493 of the active site, which is not present in OXC enzymes. Mutations to alanine, glutamine, or lysine resulted in a substantial decrease of AcHACL activity, demonstrating the importance of the glutamate side chain for the cleavage of 2-HIB-CoA. Moreover, a large C-terminal lid domain seems to restrict the size of the substrate 2-hydroxyacyl residue in AcHACL. The latter finding opens a starting point toward different substrate specificities for the future enzyme engineering of new synthase reactions.

## Results and discussion

### AcHACL shows a compact dimer of dimers structure

**Figure 2:**
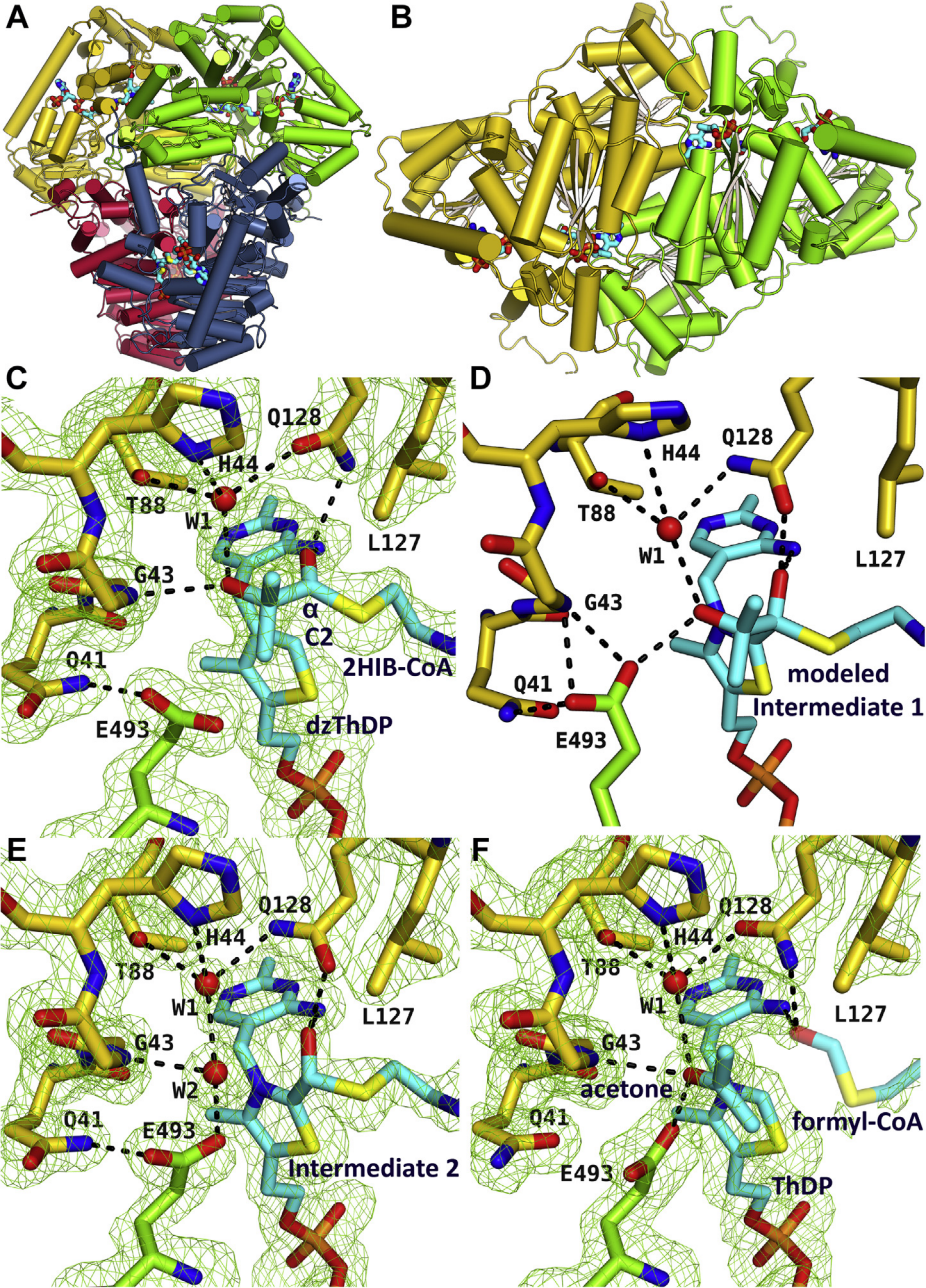
**AcHACL enzyme structures trapped in different ligand-bound states.***A*, the tetrameric AcHACL enzyme architecture can be described as a dimer of dimers. *B*, the top view of one dimer shows that the active sites are located between two protein chains. *C*–*F*, zoom into the active site of (*C*) the substrate-bound crystal structure, which was obtained in the presence of the inactive cofactor dzThDP, (*D*) the first intermediate bound modeled state in which cofactor ThDP is covalently bound to 2-HIB-CoA, (*E*) the second intermediate bound crystal structure and (*F*) the product-bound enzyme structure. The ligands are shown in *light blue*. The ligand interacting enzyme residues are colored in *green* and *yellow* according to the protein chain from which they originate. Omit electron density maps are shown for the crystal structures at 2.5 σ level. Formyl-CoA is depicted in dim colors because the formyl-cysteamine-β-alanyl residue is not well defined in the electron density. Hydrogen bonds between the ligands and the protein are shown as *black dashed lines*.

We have solved various cocrystal structures of WT and mutant AcHACL in the presence of the substrate 2-HIB-CoA and the active and inactive cofactors ThDP and dzThDP, respectively (Table 1). The AcHACL structure was solved by molecular replacement using the acetolactate synthase (ALS) from *Bacillus subtilis* (BsALS, PDB ID: 4RJI) as a molecular replacement model. AcHACL is the first published HACL crystal structure and forms a compact tetrameric assembly such as the OXC and ALS proteins (8, 20, 21). Likewise, the tetramer can be described as a dimer of dimers (Fig. 2*A*). This arrangement is also conserved in other ThDP-dependent enzymes (22) where each dimer forms two equivalent active sites for the binding of the cofactor and the substrate/intermediate at the dimeric interfaces (Fig. 2*B*). Surprisingly, in contrast to the structurally related OXC (8), no ADP is bound in the AcHACL structure, although it was present in the crystallization solution. The cognate binding site is occupied in AcHACL by the side chains of residues R107, R230, E256, R288, D312, and E316 (Fig. S1). Therefore, ADP is not required for the stabilization of the functional enzyme conformation as previously suggested for OXC (8). However, as the ADP-binding site is conserved in RuHACL (PDB ID: 6XN8, Fig. S1) and in the recently modeled structure of HsHACL1 (AF-Q9UJ83-F1) (23), these enzymes may share the ADP feature with OXC.

**Table 1:** Crystallographic data and refinement statistics

	WT AcHACL 2-HIB-CoA + dzThDP	E493Q mutant 2-HIB-CoA + dzThDP	E493A mutant 2-HIB-CoA + dzThDP	WT AcHACL intermediate/products
Data collection				
Beamline	DLS I03	DLS I03	DLS I03	DLS I03
Space group	C222_1_	C222_1_	C222_1_	P6_5_22
Cell dimensions				
a, b, c (Å)	104.0/146.1/174.7	103.7/145.5/174.2	103.9/146.7/174.4	116.2/116.2/312.1
α, β, γ (°)	90.0/90.0/90.0	90.0/90.0/90.0	90.0/90.0/90.0	90.0/90.0/120.0
Resolution (Å)	87.37–1.55 (1.75–1.55)^a^	87.10–1.76 (1.96–1.76)	87.21–1.63 (1.78–1.63)	100.66–1.64 (1.84–1.64)
R_merge_ [%]	11.4 (153.7)	10.3 (138.2)	10.3 (162.4)	18.9 (153.9)
R_pim_ [%]	3.5 (47.3)	3.1 (43.3)	3.1 (48.1)	3.0 (24.9)
<I/σI>	10.1 (1.9)	15.0 (1.7)	14.2 (1.5)	15.3 (1.7)
Completeness (%)^b^	89.4 (60.9)	95.4 (71.5)	94.8 (69.0)	96.8 (82.5)
Redundancy	11.4 (11.3)	11.9 (11.1)	11.8 (12.2)	40.1 (38.7)
CC(1/2)	0.999 (0.644)	0.999 (0.736)	0.999 (0.686)	0.999 (0.878)
Refinement				
R_work_/R_free_	16.0/19.4	14.7/18.5	15.4/18.6	14.5/17.5
Ramachandran plot				
Most favored [%]	97.2	97.4	97.3	98.0
Allowed [%]	2.8	2.6	2.7	2.0
Disallowed [%]	0.0	0.0	0.0	0.0
No. atoms				
Protein	8616	8627	8625	8786
Water	1017	1003	969	1049
Ligand	163	164	163	255
*B*-factors				
Protein	22.0	28.3	23.2	20.2
Water	19.9	33.7	30.6	29.1
Ligands	20.0	26.3	20.6	48.0
R.m.s. deviations				
Bond lengths (Å)	0.0142	0.0137	0.0133	0.0137
Bond angles (°)	1.8530	1.8490	1.7780	1.7590
PDB ID	7PT1	7PT2	7PT3	7PT4

a Values in parentheses are for the highest-resolution shell.

b Ellipsoidal completeness.

### 2-HIB substrate residue specifically interacts with active site residue Q128 and a central water molecule

To gain insights into substrate 2-HIB-CoA binding, we have solved an AcHACL crystal structure in the presence of the inactive cofactor dzThDP to 1.55 Å resolution. Berthold *et al.* (9) have already shown for OfOXC that the acyl-CoA substrate (2-HIB-CoA in AcHACL) can be trapped in the enzyme by replacing ThDP with dzThDP, an efficient inhibitor of ThDP-dependent enzymes (24). In AcHACL, the substrate 2-HIB-CoA mainly interacts through its CoA moiety resulting in multiple interactions with, *e.g.*, residues Q255, R273, S274, R362, R417, K564, and D561 of one protein chain (Fig. S2). The 2-HIB acyl residue, on the other hand, is well positioned in the active site (Fig. 2*C*) by hydrogen bonds of its carbonyl (Cα) oxygen with atom N4′ of the cofactor and with the δ amino group of residue Q128 from the adjacent protein chain within the same dimer. This brings the Cα atom and the thiazolium C2 atom in catalytic proximity of about 3.3 Å (Fig. 2*C*). Furthermore, a central water molecule (W1) forms hydrogen bonds with the tertiary alcohol oxygen atom of 2-HIB as well as with active site amino acids H44, T88, and Q128. In the obtained AcHACL structure, all substrate-binding pockets of the tetramer are occupied with 2-HIB-CoA, indicating that the asymmetry between paired active sites observed in the pyruvate dehydrogenase E1 component (25) or other ThDP-dependent enzymes (26) is not found in AcHACL. The symmetry found in AcHACL was also observed for OfOXC (9), suggesting that in both enzymes all reactive sites of the tetramer might be active at the same time.

### Covalently bound intermediate maintains hydrogen bonding with Q128 and gain proximity to E493

An AcHACL crystal structure of an intermediate state was obtained by cocrystallization with the substrate 2-HIB-CoA and the cofactor ThDP. The crystals were soaked for an additional 2 h with fresh substrate and cofactor before freezing. Diffraction data were collected to 1.64 Å resolution. Interestingly, the active sites of the tetramer contain ligands in different reaction states. One dimer within the tetrameric assembly clearly shows a covalently bound postcleavage intermediate, whereas the products formyl-CoA and acetone are bound to the other dimer within the same tetramer.

The postcleavage intermediate bound crystal structure represents the second intermediate state in the catalytic cycle after 2-HIB-CoA Cα-Cβ cleavage and release of the first reaction product acetone. For modeling the first intermediate, we added 2-hydroxyisopropyl to the Cα-atom of the second intermediate structure and performed molecular dynamics simulations. This resulted in the tetrahedral precleavage complex (Fig. 2*D*). The Cα oxygen of both intermediate bound states still interacts with residue Q128, although it became a hydroxyl group during covalent bond formation, hence, interacts now with the Q128 carbonyl oxygen of the side chain instead of the amino group. The formation of the covalent bond between the substrate Cα and the cofactor C2 leads to a translation of the substrate acyl toward the cofactor that causes a shift of the protein loop with residue Q128 in order to maintain the interaction. The glutamine residue is conserved in ALS (Q124 in BsALS) as well as in RuHACL (Q113). In AcHACL, the N4′ atom within the aminopyrimidine ring of ThDP maintains the interaction with the Cα oxygen as well. Interestingly, while still interacting with water molecule W1 as seen for the substrate-bound structure (Fig. 2*C*), the 2-HIB Cβ hydroxyl group forms now a hydrogen bond with the side chain of amino acid E493, which is part of an α-helix that is only present in AcHACL and ALS but not in OXC. Compared with the substrate state (Fig. 2*C*) and first intermediate complex (Fig. 2*D*), an additional water molecule (W2) has entered the active site in the second intermediate bound structure. This water fills the space that was previously occupied by the Cβ hydroxyl group of the 2-HIB residue (Fig. 2*E*). In line with this, the second water molecule forms hydrogen bonds with W1 and the side chain of E493. In addition, it interacts with the peptide backbone of G43. A similar situation with two well-coordinated water molecules in the active site is found in the second covalent intermediate structure of OfOXC (9).

### Formyl-cysteamine-β-alanyl residue is not well coordinated in product bound structure

While the dimer with the second covalently bound intermediate is well-resolved, the electron density of the CoA thioester within the other dimer is not well defined beyond the pantoyl moiety. The additional electron density close to the ThDP cofactor was interpreted as an acetone molecule (Fig. 2*F*). Consequently, we concluded that this dimer is occupied by the AcHACL reaction products formyl-CoA and acetone. In contrast, the OfOXC cocrystal structure with formyl-CoA shows an unambiguous electron density for the thioester terminus. This is most likely due to multiple interactions of the formyl Cα oxygen with residues Y483 and S553 (OfOXC numbering) as well as with a water molecule, guaranteeing appropriate orientation within the active site (9). In AcHACL, however, only hydrogen bonds with cofactor N4′ and the Q128 side chain might occur resulting in increased flexibility of the thioester terminus and, hence, explain the less defined electron density. In contrast, the first lyase reaction product, acetone, could be trapped in the same dimer (Fig. 2*F*). Its carbonyl oxygen atom still forms a hydrogen bond with the side chain of E493 as previously observed in the modeled first intermediate structure for the equivalent Cβ oxygen atom (Fig. 2*D*).

### Less active E493 mutants show equal or improved substrate binding

Kinetic parameters for the 2-HIB-CoA lyase reaction were obtained at optimal 37 °C and pH 7.2. The *K*_*m*_ and *k*_*cat*_ values for the WT enzyme are close to 120 μM and 1.3 s^−1^, respectively, corresponding to a catalytic efficiency of about 11 s^−1^ mM^−1^ (Table 2 and Fig. S6). Besides the conserved active site residue Q128, amino acid E493 seems to play an important role for the catalytic mechanism. Therefore, we have produced enzyme mutants E493A, E493Q, and E493K to study the function of the glutamic acid side chain for the catalytic reaction. All mutations resulted in a substantial decrease of the *k*_*cat*_ values, confirming the catalytic role of E493 in AcHACL. Compared with the WT enzyme, the E493A and E493Q mutants exhibit a 10- and 50-fold reduction of the 2-HIB-CoA cleavage rates, respectively (Table 2, Figs. S7 and S8). Moreover, AcHACL activity of the E493K mutant is even ≥12,000 times diminished (below the detection limit). Surprisingly, the *K*_*m*_ values for the mutant enzymes do not show the same tendency as the conversion rates (Table 2). While the *K*_*m*_ of the E493Q mutant is comparable to the WT value, the *K*_*m*_ of E493A is five times lower, indicating improved catalytically competent 2-HIB-CoA accommodation in the substrate-bound structure.

**Table 2:** Kinetic parameters of WT AcHACL and mutant enzymes obtained at optimal pH 7.2 and 37 °C for the cleavage of 2-HIB-CoA to formyl-CoA and acetone

Enzyme variant	*K*_*m*_ (μM)	*V*_*max*_ (nmol min^−1^ mg^−1^)	*k*_*cat*_ (s^−1^)	*k*_*cat*_/*K*_*m*_ (s^−1^ mM^−1^)
WT	120 ± 12	1200 ± 20	1.3 ± 0.04	11 ± 1
E493A	21 ± 4	98 ± 4	0.11 ± 0.004	5.1 ± 0.9
E493Q	79 ± 7	25 ± 1	0.03 ± 0.001	0.34 ± 0.03
E493K	N/A^a^	≤0.1	N/A	N/A

Values given refer to kinetic parameter and SD derived from kinetic plots (Figs. S6–S8) by applying nonlinear regression analysis and the Michaelis–Menten equation (see the Supporting information for further details).

a N/A, not applicable.

### 2-HIB accommodation is suboptimal in the E493 and Q493 enzymes

**Figure 3:**
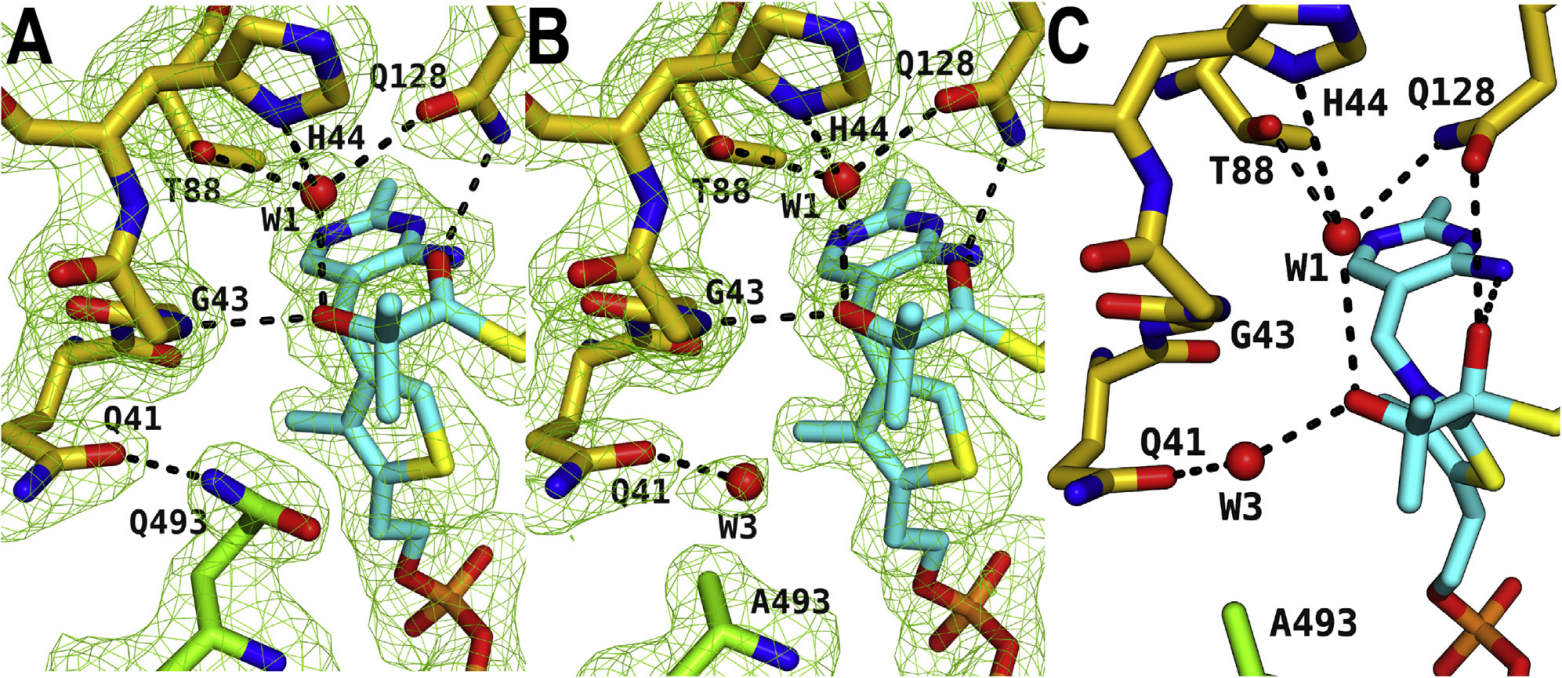
**AcHACL mutant enzyme structures.** Zoom into the active site of (*A*) the substrate-bound crystal structure of the AcHACL mutant E493Q, (*B*) the substrate-bound crystal structure of the enzyme mutant E493A, and (*C*) the first intermediate bound modeled state of the AcHACL mutant E493A. The color scheme is the same as in Figure 2. Omit electron density maps are shown for the crystal structures at 2.5 σ level.

To study the underlying reason for the deviating *K*_*m*_ values, we have determined the crystal structures of the enzyme mutants E493Q and E493A in the presence of the substrate 2-HIB-CoA and inactive cofactor dzThDP to 1.76 Å and 1.63 Å resolution, respectively (Fig. 3, *A* and *B*). Moreover, we have performed molecular dynamics simulations of the first covalent intermediate state in the presence of the alanine mutant (Fig. 3*C*). With rmsd values of about 0.1 Å, the overall structures and the substrate-binding modes are identical in the mutants and the WT enzyme. As outlined above for the WT structure, the majority of substrate interactions with the protein are formed by the CoA moiety, and the orientation of the acyl residue in the dzThDP-bound structures is identical as well. As expected, the conformation of the Q493 residue of the mutant is almost identical to the one of WT E493 and interacts likewise with Q41 (Fig. 3*A*). In the E493A mutant, the glutamic acid side chain position in the WT is occupied by a water molecule (W3 in Fig. 3*B*). Interestingly, W3 also mimics the WT E493 role and interacts with the Cβ hydroxyl group in the modeled first intermediate complex (Fig. 3*C*). Thus, W3 can compensate for the missing side chains of E493 and Q493 in the E493A mutant. A closer inspection revealed that substrate binding is suboptimal in the WT and the E493Q mutant, since one methyl group of the 2-HIB residue binds very close to the side chain of E493 (2.9 Å; Fig. 2*C*). In contrast, the 2-HIB methyl groups in the E493A mutant structure are further away from the W3 water molecule and the A493 side chain, thereby allowing a better accommodation of the substrate (Fig. 3*B*).

### E493 acts as an acid/base catalyst during 2-HIB cleavage and Cα-carbanion protonation

**Figure 4:**
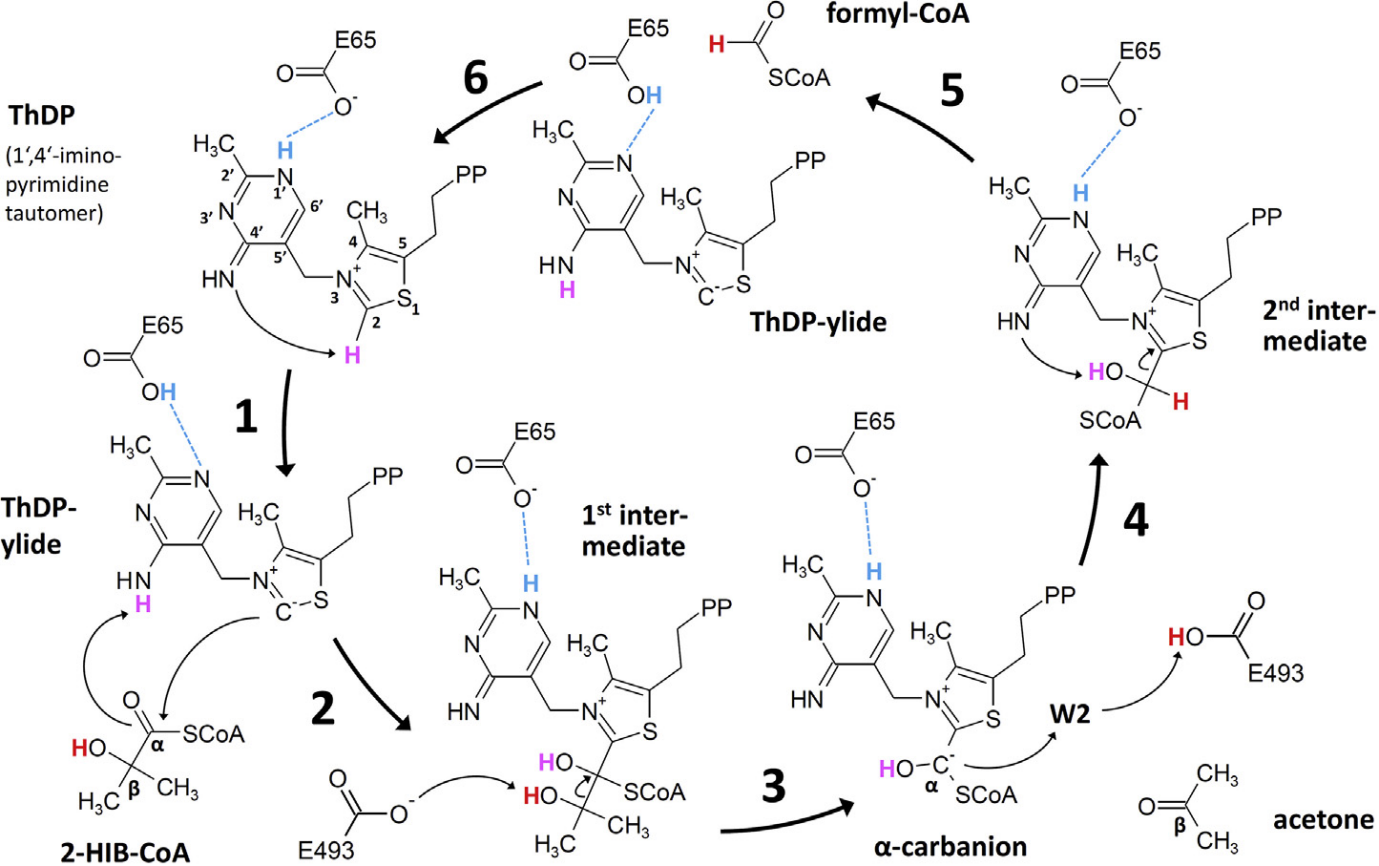
**Proposed catalytic cycle for the WT AcHACL-catalyzed reaction**.

We have determined various crystal structures of AcHACL in complex with the substrate 2-HIB-CoA, the second covalent intermediate as well as products formyl-CoA and acetone. Together with the molecular dynamics simulations of the first covalently bound intermediate state, we are able to describe the full catalytic cycle for the WT enzyme (Fig. 4). In step 1, the conserved glutamic acid E65 side chain interacts with atom N1′ of the ThDP aminopyrimidine ring (Fig. S2), thereby enabling its tautomerization to the imino form (27). This allows the N4′ atom to abstract a proton from the thiazolium C2 atom resulting in the formation of the nucleophilic ThDP-ylide. After the nucleophilic attack of the thiazolium C2 atom on the substrate Cα atom (step 2), the 2-HIB carbonyl oxygen can abstract a proton from the N4′ atom, which is back in the amino form and still forms an interaction with Q128. This first covalently bound intermediate state is described by the molecular dynamics simulations (Fig. 2*D*). Importantly, the tertiary hydroxyl group of 2-HIB forms a hydrogen bond with E493. This active site residue is well positioned through hydrogen bonding with the side chain of Q41 and the protein backbone of residue G43. We hypothesize that the side chain of E493 abstracts the proton from the tertiary alcohol during the following cleavage of the 2-HIB Cα-Cβ bond (step 3), resulting in the formation of the Cα-carbanion intermediate and the release of the carbonyl product acetone. In step 4, the Cα-carbanion is protonated, most likely *via* water molecule W2 in the second intermediate bound crystal structure (Fig. 2*E*). This water molecule is in direct contact with residue E493 that can transfer back the previously accepted proton from the tertiary alcohol oxygen. Accordingly, the 50-fold reduction in the cleavage rate found for the E493Q enzyme mutant can be partially explained by the low acidity of the amide compared with the E493 γ carboxylic group. Hence, the conjugate Q493 amide anion is rarely available to accept a proton from the Cβ hydroxyl group of 2-HIB in step 3. However, in case of glutamate as exclusive acid/base catalyst, typically, a mutation to glutamine would have a more dramatic effect, *e.g.*, a >5000-fold reduction in conversion rates (28). Therefore, alternative proton transfer routes might be present, possibly, through poorly coordinated water molecules. Although not present in the electron density, this water could well accommodate in close proximity to the 2-HIB residue in the mutant structure (Fig. 3*A*). In the E493K mutant, on the other hand, the ε amino group of lysine might be too basic to support efficient proton exchange. In addition, the larger lysine side chain most likely impedes substrate binding. Consequently, this mutation results in a complete loss of lyase activity. In contrast, the only tenfold rate reduction observed in the alanine mutant of AcHACL is somewhat surprising, as a >25,000 times decrease is expected (28). As proposed for the E493Q mutant, water molecules might be involved in proton transfer. In line with this, water molecule W3 is well coordinated in the active site within the mutant enzyme (Fig. 3*B*) and may substitute for the E493 side chain by acting as an acid/base catalyst during Cα-Cβ bond cleavage and Cα-carbanion protonation, albeit at much lower efficiency. Finally, the imino N4′ atom abstracts a proton from the Cα hydroxyl group and formyl-CoA is released as second product (step 5). The cofactor ThDP may be restored from the ylide by proton transfer from E65 through atoms N1′ and N4′ of the aminopyrimidine to atom C2 of the thiazolium ring (step 6), thereby completing the proton movements within the catalytic cycle.

### The C-terminal lid as well as the active site amino acids L127 and I492 determines the substrate acyl size

**Figure 5:**
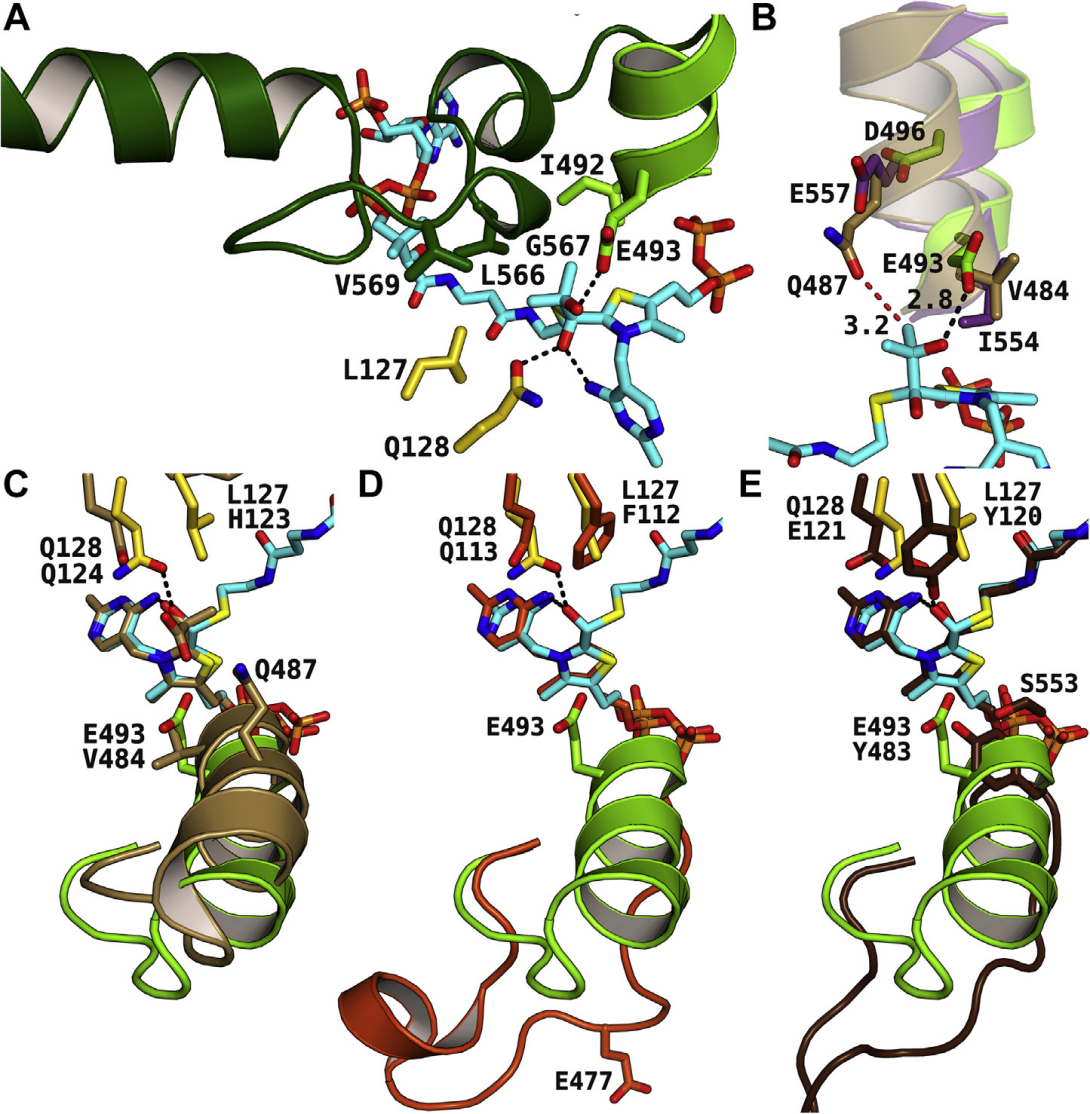
**Active site architecture of AcHACL and enzyme homologs.***A*, the size of the active site of AcHACL is restricted by the E493-containing α-helix (*light green*) and the C-terminal lid domain (*dark green*). The enzyme residues interacting with the 2-HIB residue are colored in *green* and *yellow* according to the protein chain from which they originate. The modeled first intermediate is shown in *light blue*. Hydrogen bonds between the 2-HIB residue and the protein are shown as *dashed black lines*. *B*, superposition of the crystal structures of AcHACL (*green*) and BsALS (*beige*, PDB ID: 4RJI) with the AlphaFold model of HsHACL2 (*violet*, AF-A1L0T0-F1) shows that the active site α-helix is in a closed conformation for all three structures. Residue E493 forms an interaction with the 2-hydroxyl group of the first intermediate (*light blue*) in AcHACL, whereas HsHACL2 and BsALS have an isoleucine and valine residue, respectively, at this position that prevents H-bonding interactions. However, in HsHACL2 and several related enzymes, a glutamic acid is present three residues later (one helix turn) in the sequence within the α-helix (Fig. S3), which is possibly involved in substrate interactions. In BsALS, the enzymatic mechanism is different, but the glutamine residue Q487 is located next to the AcHACL intermediate within the superimposed structures, thereby illustrating a possible interaction mechanism for HACL enzymes that is highlighted as *dashed red line*. Values refer to atomic distance in Å. *C*–*E*, zoom into the active site of the superposition of the second intermediate bound AcHACL crystal structure with the crystal structures of (*C*) BsALS in complex with a 2-lactyl bound ThDP intermediate (PDB ID: 4RJK), (*D*) RuHACL in complex with the cofactor analog TzDP (PDB ID: 6XN8) and (*E*) OfOXC with covalently bound second intermediate (PDB ID: 2JI7). The ligands and ligand interacting amino acids are colored in *beige*, *orange*, and *brown*, respectively, for the structural homologs.

With rmsd values of 2.4 Å, a DALI search results in OfOXC and the recently deposited RuHACL structure as the closest structural homologs of AcHACL. The structures of RuHACL and OfOXC, on the other hand, are structurally related to each other with an rmsd value of 1.8 Å. Accordingly, AcHACL possesses structural features that are not present in OfOXC or in RuHACL, including a large C-terminal domain spanning over residues 556 to 590 that closes the active site upon substrate binding (Fig. 5*A*). Moreover, residues 564 to 575 break out from an α-helix (residues 560–563 and 576–587) and fold over the 2-HIB residue (Fig. 5*A*) as well as interact with the neighboring protein chain within the same dimer. Together with the active site residues L127 and I492, the C-terminal helical breakout restricts substrate sizes to short-chain acyl residues (C4, C5). RuHACL, HsHACL1, and HsHACL2, on the other hand, have much shorter C-termini. Despite the lack of structural information, the shorter C-termini cannot restrict the substrate acyl size. Consequently, all these lyases are able to catalyze the cleavage of much larger CoA thioesters (at least C18 acyl residues).

### The E493-bearing α-helix is a common feature in HsHACL2-like enzymes

The putative catalytic residue E493 is located within an 11 amino acid long α-helix in AcHACL (Fig. 5, *A* and *B*). Likewise, the modeled structure of HsHACL2 (AF-A1L0T0-F1) (23) as well as of closely related lyases possesses a similar α-helix, and the corresponding sequence segment can be well aligned with the one of AcHACL (Fig. S3). The HsHACL2 E557-containing α-helix, for example, forms a closed conformation similar to the AcHACL α-helix, and the side chain is oriented toward the active site (Figs. 5*B* and S4). Despite being one helical turn further away from the intermediate, we speculate that the α-helix can rotate even further toward the active site due to a substantially shorter C-terminus in HsHACL2 (Fig. S4) that would bring residue E557 much closer to the Cβ-hydroxyl group. The α-helix is conserved in ALS enzymes as well, but not the residue with the carboxylic acid side chain (Fig. S3). Consistent with a different catalytic mechanism and the need to bind a second pyruvate substrate after formation of the covalent intermediate, a carboxylic acid side chain is not required, and the α-helix is not involved in substrate or intermediate interactions. In BsALS, which was used as a molecular replacement model, the corresponding residue to E493 in AcHACL is V484, and the only residue harboring a carboxylic side chain within the α-helix is aspartic acid D482. Both residues, D482 and V484, are oriented away from the bound intermediate (PDB ID: 4RJK, chain H) (Figs. S3 and 5*C*). Interestingly, in the superposition with AcHACL, the α-helix in BsALS is orientated closer to the active site and the Q487 residue corresponding to E557 in HsHACL2 (Fig. S3) would be in appropriate distance to the AcHACL 2-HIB-CoA intermediate (Figs. 5*B* and S4). This is in support of our hypothesis that E557 in HsHACL2, although one helix turn later than E493 in AcHACL could be in catalytic distance to the substrate. Furthermore, the E557 and E493 residues are well conserved in HsHACL2-related (Fig. S3) and AcHACL-related (Fig. S5) enzymes, respectively. Interestingly, a glutamic acid is present on a comparable sequence segment in RuHACL as well (E477, RuHACL numbering), although again three residues later than in AcHACL (Fig. S3). The RuHACL structure (PDB ID: 6XN8), which has not yet been published, was deposited in the PDB in the open state because no CoA thioester substrate is bound and the C-terminus, which traps the substrate in its active site is unstructured. Consequently, the glutamic acid E477 containing loop is flipped away from the active site (Fig. 5*D*), most likely due to missing substrate and C-terminal interactions. However, even when interacting with a substrate, it is unlikely that RuHACL forms a corresponding α-helix, as the respective region contains four proline residues (Fig. S3). In contrast, all AcHACL crystal structures were crystallized in closed substrate, intermediate, and product-bound states and show the α-helix adjacent to the C-terminal lid. Furthermore, the modeled structure of HsHACL1 (23) shows a deviating α-helix (Fig. S3). Consequently, although several candidate glutamic and aspartic acid residues are present in the latter enzyme, an active site amino acid playing the catalytic role proposed for E493 in AcHACL cannot be unambiguously assigned.

In summary, the α-helix containing the carboxylic acid side chain which interacts with the 2-hydroxyacyl residue of the CoA thioester substrate appears to be a common feature of AcHACL and HsHACL2-like enzymes. In RuHACL and HsHACL1, on the other hand, the secondary structure is different and the assignment of a glutamic or aspartic acid residue corresponding to E493 in AcHACL is uncertain.

### Reflections on design of tailored OXC and HACL enzymes

In OXC, the decarboxylation step does not require a proton transfer (Fig. 1), and in line with this, an α-helix with a corresponding active site amino acid acting as an acid/base catalyst is not present. In OfOXC, a tyrosine residue (Y483) is located at a similar position as the catalytic E493 in AcHACL and forms together with S553 hydrogen bonding interactions with the carboxylic group of the oxalyl residue (Fig. 5*E*). Accordingly, the tyrosine and serine residues were replaced by phenylalanine and glycine, respectively, in the quadruple MeOXC mutant (Y497F and S568G) to improve the condensation reaction of formyl-CoA with formaldehyde, since the latter as well as the resulting glycolyl-CoA do not contain a carboxylic group anymore (10). Furthermore, the substrate Cα oxygen interacting glutamine residue found in HACL and other ThDP-dependent enzymes (Q113 and Q128 in RuHACL and AcHACL, respectively) is replaced by a conserved glutamic acid in WT OXC (E121 and E135 in OfOXC and MeOXC, respectively). The preceding tyrosine directly interacts instead with the substrate oxalyl residue (Y120 in OfOXC, Fig. 5*E*). The mutation of the glutamic acid to a glycine residue in MeOXC increased the rate of the synthase reaction, most likely because the tyrosine side chain is given more space for optimal interaction with the intermediate Cα-hydroxyl group (10). Unfortunately, substrate or covalently bound intermediate structures have not yet been solved for the optimized MeOXC mutant and only a structure in open conformation has been deposited (PDB ID: 7B2E). Nevertheless, the superposition of AcHACL with OfOXC (Fig. 5*E*) clearly indicates that for transferring the HACL reaction to OXC, thus far, focus has been only laid on mutations to avoid unfavorable interaction during substrate accommodation. Consequently, the resulting MeOXC mutant is quite promiscuous, as carbonyl compounds of varying size (from formaldehyde to phenylacetaldehyde) serve as substrate (10). Furthermore, since the MeOXC mutations E135G, Y497F, and S568G do not provide the acid/base catalyst required for reverse steps 3 and 4 in the HACL catalytic cycle (Fig. 4), proton transfer is likely mediated by water molecules. And the latter cannot be coordinated by the mutated positions, as only nonpolar side chains were added to the active site. In summary, to tap the full potential of the HACL reaction in biotechnology, additional manipulations might be required to increase both substrate specificity and conversion rate. The present study on AcHACL structure and underlying reaction mechanism appears to be quite helpful for this endeavor.

## Conclusion

The elucidated AcHACL enzyme structures reveal important details about the mechanism of the catalyzed 2-hydroxyacyl-CoA cleavage reaction. These could enable the structure- and mechanism-guided design of improved enzyme variants for the biotechnologically interesting condensation of formyl-CoA with aldehydes or ketones. All determined AcHACL crystal structures are in a closed state with bound substrates, intermediates, or products. Correlating with the preference for short-chain substrates, AcHACL possesses an extended C-terminal lid domain not present in other HACL enzymes or OXC. Since larger acyl substrates will clash with the C-terminus and its helical breakout, the latter determines substrate specificity to short-chain 2-HIB (C4) and 2-hydroxy-2-methylbutyryl (C5) residues. In addition, the active site amino acids L127 and I492 limit the substrate size. Therefore, modifying the lid residues as well as positions 127 and 492 may increase specificity toward even smaller substrates of interest, such as glycolaldehyde and formaldehyde. The E493-containing α-helix found in AcHACL functions as movable and presumably important acid/base catalyst to the active site. Most likely, this structural feature is conserved in other HACL enzymes, albeit the catalytic glutamic acid might be replaced by aspartic acid in some cases. With respect to the mechanism, among all AcHACL enzyme variants tested, the WT E493 residue appears to be the best answer for balancing the tradeoff between optimal binding mode and catalytic function. Considering the proposed catalytic role of E493 in proton transfer from the Cβ hydroxyl to the Cα-carbanion, it is quite plausible that all mutants tested show lower 2-HIB-CoA conversion rates. This proton transfer specific for the 2-hydroxyacyl-CoA cleavage is not present in OXC catalysis and has not been considered in the thus far established mutations for optimizing the condensation reaction with formyl-CoA and short-chain carbonyl compounds in MeOXC. In the latter enzyme as well as in the E493A and E493Q mutants of AcHACL, coordinated or noncoordinated active site water molecules may act as acid/base catalysts.

## Experimental procedures

### Cloning, expression, and purification of recombinant proteins

The AcHACL WT gene sequence was previously cloned into the pASG-IBA43 vector (IBA Lifesciences) containing an N-terminal His-tag and a C-terminal Strep-tag (19). AcHACL was expressed in *Escherichia coli* Lemo21 (DE3) (NEB). The cells were grown in Terrific Broth medium at 37 °C until an optical density at 600 nm of 0.6 and then cooled down to 20 °C before induction with 200 μg l^−1^ anhydrotetracycline. After cultivation for another 16 h at 20 °C, cells were lysed in 20 mM Tris pH 8.0, 300 mM NaCl, 3 mM MgCl_2_ and DNase by sonication. The protein was purified with Ni-affinity and size-exclusion chromatography and eluted into 20 mM Tris pH 7.5, 150 mM NaCl and 3 mM β-mercaptoethanol. AcHACL mutant enzymes were purified using the same protocol. For determining kinetics with the WT enzyme, cells were alternatively disrupted in 100 mM potassium phosphate, 10% glycerol, pH 7.2 using a mixer mill at 30 s^−1^ for 30 min and glass beads as described previously (29). After Ni-affinity chromatography, the purified enzyme was transferred into conservation buffer (100 mM potassium phosphate, 10% glycerol, pH 7.2 supplemented with 1 mM ThDP and 5 mM MgCl_2_) with PD-10 desalting column (GE Healthcare). Both purification protocols described above resulted in AcHACL preparations with comparable activities, although for prolonged storage as cryostocks, the conservation buffer appeared to be superior and several cycles of freezing and defrosting did not change lyase reaction rates. Mutant enzymes were obtained by site-directed mutations using primers E493Q_for: CAGCGCTACGACCAGGCCGAGA, E493A_for: GCGCGCTACGACCAGGCCGAGA, E493K_for: AAGCGCTACGACCAGGCCGAGA and rev: GATGTTCCAGGCGCGGTTGTT.

### 2-HIB-CoA and dzThDP synthesis

2-HIB-CoA was synthesized from 2-HIBA and CoA according to an established protocol *via* the corresponding thiophenol thioester intermediate (29). The dzThDP used in this study was synthesized from the corresponding deazathiamine *via* phosphorylation. 3-Deazathiamine was prepared based on the procedures described by Leeper and coworkers (30) with minor modifications. Then, the pyrophosphate was prepared according to Zhao’s procedures (31) with necessary changes in purification methods. Briefly, the hydroxyl group of 3-deazathiamine was converted to the corresponding tosylate, which set the stage for an S_N_2 substitution with tris-(tetra-n-butylammonium) hydrogen pyrophosphate in acetonitrile at room temperature (32). The dzThDP was purified by anion exchange chromatography in order to prevent the formation of mono phosphate. Proton and carbon NMR spectra were recorded on a Bruker Avance III (600 MHz) spectrometer. High-resolution mass spectra were run on Waters LCT Premier machine. Reactions were monitored using analytical ultrahigh pressure liquid chromatography on a Waters Acquity SQD (BEH C18 column 1.7 μm 2.1 × 100 mm, flow rate 0.3 ml min^−1^, 6 min run with a gradient of 1–20% acetonitrile and water both with 0.01% formic acid). Anion exchange was performed on an ISCO CombiFlash chromatography system using a column that was prepacked with DEAE-Sephacel (Sigma). Analytical TLC was performed on commercial Merck glass plates, coated to a thickness of 0.25 mm with Kieselgel 60 F254 silica. Finally, lyophilization gave the dzThDP as a white powder (ammonium salt 24 mg, 60%). ^1^H NMR (600 MHz, D_2_O) δ 7.46 (s, 1H), 6.73 (s, 1H), 3.94 (dt, coupling constant J = 6.8, 6.6 Hz, 2H), 3.54 (s, 2H), 2.97 (t, J = 6.5 Hz, 2H), 2.30 (s, 3H), 1.89 (s, 3H); ^13^C NMR (150 MHz, D_2_O) δ 163.4, 162.8, 148.6, 136.4, 135.5, 133.8, 119.7, 114.0, 65.9, 28.9, 27.4, 22.3, 11.4; ultrahigh pressure liquid chromatography (electrospray ionization) calculated for C_13_H_20_N_3_O_7_P_2_S (M + H), 424.0497; found, 424.0502.

### Crystallization and structure determination

The size-exclusion chromatography purified proteins (WT and enzyme mutants E493Q as well as E493A) were concentrated to 10 mg ml^−1^ and sitting drop cocrystallization trials with 1 mM 2-HIB-CoA, 5 mM ADP, 5 mM MgCl_2_ and 5 mM dzThDP were set up with a Mosquito crystallization robot (sptlabtech) using SWISSCI 3-lens low profile crystallization plates. WT AcHACL and the mutants E493Q as well as E493A yielded crystals under the PACT conditions B3 (25% PEG 1500, 0.1 M MIB buffer pH 6.0), B4 (25% PEG 1500, 0.1 M MIB buffer pH 7.0) and A3 (25% PEG 1500, 0.1 M SPG buffer pH 6.0), respectively. Crystals of an intermediate bound state grew in 2 M ammonium sulfate and 0.1 M HEPES pH 7.0 in the presence of 1 mM 2-HIB-CoA, 5 mM ADP, 5 mM MgCl_2_ and 5 mM ThDP. Before harvesting, crystals were soaked for additional 2 h with fresh 2-HIB-CoA as well as ThDP. Diffraction data were collected on beamline I03 at the Diamond Light Source (Didcot) and automatically processed with STARANISO (33) on ISPyB. The structure was solved within CCP4 Cloud by molecular replacement with MoRDa (34) using PDB ID: 4RJI as search model. Model building was performed in Coot (35), and the structure was refined with Refmac 5 (36). MolProbity (37) was used to evaluate the final model and PyMOL (Schrödinger, LLC) for protein model visualization. Data and Refinement statistics are summarized in Table 1. A search for structural protein homologs and calculation of rmsd values were performed with the DALI server (38).

### HPLC-based AcHACL activity assay

Kinetic parameters for the 2-HIB-CoA lyase reaction were determined with the AcHACL WT enzyme (Fig. S6) at optimal temperature and pH of 37 °C and 7.2, respectively, using a discontinuous HPLC-based assay slightly modified from a previously described method (29). Briefly, the enzyme was preincubated in 100 mM potassium phosphate buffer supplemented with 10% glycerol, 0.2 mM ThDP, 0.2 mM ADP, and 5 mM MgCl_2_ for 30 s. Afterward, 2-HIB-CoA was added to start the reaction. An assay sample was diluted in two volumes of stop buffer (100 mM sodium acetate, pH 3.5, 60 °C) and incubated at 60 °C for 5 min. Then, CoA thioesters were analyzed through an HPLC system equipped with a Nucleosil 100-5 C18 HD analytical column (Macherey-Nagel), 0.8 ml min^−1^ of eluent (100 mM sodium phosphate, 10 mM tetrabutyl ammonium hydrogen sulfate, 19% acetonitrile, pH 4.5) and a photodiode array detector SPD-M20A (Shimadzu). Quantification of thioesters was carried out at 260 nm. Accordingly, kinetics of the 2-HIB-CoA cleavage were analyzed for the E493A (Fig. S7), E493Q (Fig. S8) and E493K mutant enzymes. With this assay, a detection limit of 0.1 nmol min^−1^ mg^−1^ for the enzymatic activity was achieved (see also the Supporting information for further details). Kinetic parameters were calculated by nonlinear regression analysis applying the Michaelis–Menten equation using GraphPad Prism (GraphPad Software Inc).

### Molecular dynamics simulations

All simulations were carried out with the CHARMM simulation package (39, 40). The CHARMM36 force field for proteins (41, 42) and the CHARMM general force field (43) were employed. The crystallographic structure of the protein dimer, the ligand, crystal water, and the magnesium ions was prepared and solvated with CHARMM-GUI (44, 45), using a buffer of 0.1 M sodium chloride. SHAKE (46) was used to keep the TIP3P water (47) geometry rigid, using a lookup table for the solvent–solvent interactions (48). The solvated box was subjected to 2000 steps of steepest-descent energy minimization, using periodic boundary conditions and a force switching function (49) between 10 and 12 Å. The cubic box size of 132.9 Å was determined from the last half of 0.5 ns of constant pressure simulations. This equilibration was carried out with a time step of 1 fs at 300 K and 1 atm using position restraints with a force constant of 5 kcal mol^−1^ Å^−2^ on the ligand and with the Particle Mesh Ewald method (50). The Nosé–Hoover thermostat (51) was employed. To save computational costs, the system was divided into three regions. All residues within 16 Å of the ligand were selected for the active region without restraints. The heavy atoms of all residues within 12 Å of the active region were subjected to harmonic positional restraints with a force constant of 5 kcal mol^−1^ Å^−2^. All other atoms were frozen at their current positions using the CONS FIX command of CHARMM. The system was further equilibrated without periodic boundary conditions using 0.5 ns of molecular dynamics simulations at 300K with a time step of 1 fs. Finally, production simulations were performed for 5 ns at 300K with a time step of 2 fs. The presented structures are the final frame of the production phase.

## Data availability

The atomic coordinates and structure factors have been deposited in the Protein Data Bank (http://wwpdb.org/) and are available under the accession codes 7PT1, 7PT2, 7PT3, and 7PT4.

## Supporting information

This article contains supporting information.

## Conflict of interest

The authors declare that they have no conflicts of interest with the contents of this article.
