# Supplementary material for: Mechanistic details of the actinobacterial lyase-catalyzed degradation reaction of 2-hydroxyisobutyryl-CoA
Source: J Biol Chem. 2021 Dec 22;298(1):101522. doi: 10.1016/j.jbc.2021.101522 (PMC8760513; doi:10.1016/j.jbc.2021.101522)
Supplement: Supplemental Figures S1–S8 [file mmc1.pdf]

Supporting information

Mechanistic details of the actinobacterial lyase-catalyzed degradation reaction of 2-hydroxyisobutyryl-CoA

Michael Zahn[1,*], Gerhard König[1], Huy Viet Cuong Pham[2], Barbara Seroka[3], Ryszard Lazny[3], Guangli Yang[4], Ouathek Ouerfelli[4], Zenon Lotowski[3], Thore Rohwerder[2,*]

[1]Centre for Enzyme Innovation, School of Biological Sciences, Institute of Biological and Biomedical Sciences, University of Portsmouth, Portsmouth PO1 2DT, United Kingdom; [2]Department of Environmental Microbiology, Helmholtz Centre for Environmental Research - UFZ, Leipzig, Germany; [3]Faculty of Chemistry, University of Bialystok, K. Ciolkowskiego 1K, 15-245 Bialystok, Poland; [4]Organic Synthesis Core Facility, Memorial Sloan Kettering Cancer Center (MSKCC), New York, NY 10065, USA; *Correspondence: michael.zahn@port.ac.uk (M. Z.), thore.rohwerder@ufz.de (T. R.)

List of contents

Superposition of RuHACL ADP-binding site and corresponding AcHACL residues S-2

AcHACL binding sites for ligands 2-HIB-CoA, dzThDP and ThDP S-3

Structure-based sequence alignment of AcHACL with ALS, HsHACL2 and related HACL enzymes S-4

Superposition of AcHACL with BsALS and AlphaFold model of HsHACL2: C-terminal lid region and adjacent α-helix bearing a presumably catalytic glutamic acid residue S-5

COBALT alignment of AcHACL with closest BLAST hits S-6

Experimental details on enzyme assays and kinetic plots for WT AcHACL and mutants E493A and E493Q S-7

Superposition of RuHACL ADP-binding site and corresponding AcHACL residues

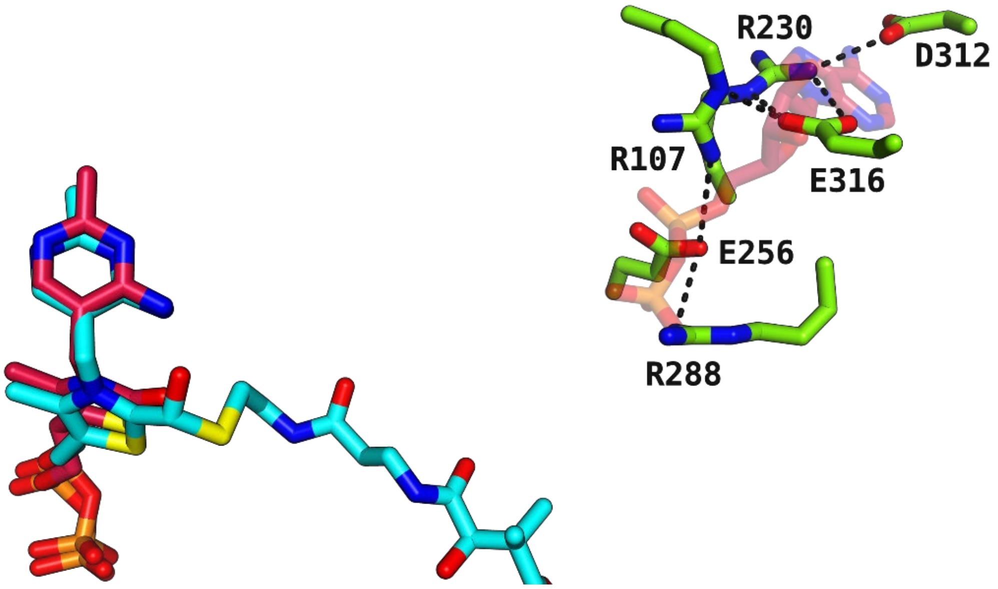

Figure S1. Superposition of the RuHACL structure (PDB ID: 6XN8; cofactor analog TzDP and ADP colored in red) with the second intermediate (light blue) bound AcHACL structure shows that several arginine and glutamic/aspartic acid AcHACL side chains (colored in green) form a network of salt bridges at the location of the RuHACL ADP binding site (RuHACL bound ADP shown half transparent), thereby preventing ADP binding in AcHACL.

AcHACL binding sites for ligands 2-HIB-CoA, dzThDP and ThDP

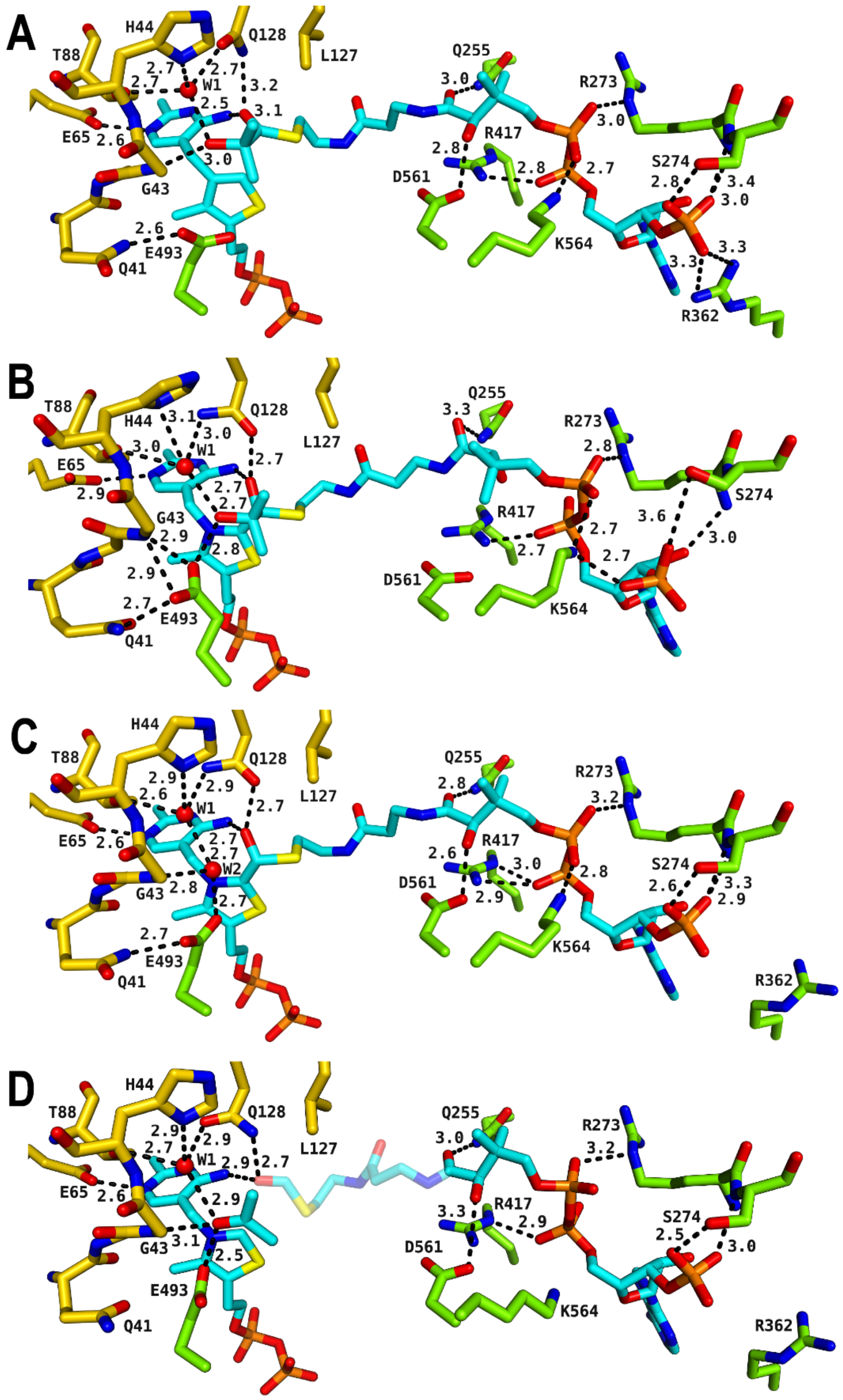

Figure S2. Binding interactions between AcHACL and (A) substrate 2-HIB-CoA as well as inactive cofactor dzThDP, (B) modeled first intermediate, (C) second intermediate and (D) product formyl-CoA and acetone as well as cofactor ThDP. The ligands are colored in light blue, the enzyme residues are colored in green and yellow according to the protein chain from which they originate. The formyl-cysteamine-β-alanyl residue of formyl-CoA is not well defined in the electron density and depicted in dim colors. Hydrogen bonds between the ligands and the protein are shown as dashed black lines with distance indicated in Å.

Structure-based sequence alignment of AcHACL with ALS, HsHACL2 and related HACL enzymes

EcALS (P08142)	470	N**N**EALGLVHQQQSLFYEQGVFA	491
BsALS (AHA79547.1)	477	N**D**STYDMVAFQQLKKYNRTSAV	498
KpALS (P27696)	473	V**D**NGYNMVAIQEEKKYQRLSGV	494
AcHACL (WP_018331913.1)	486	N**N**RAWNI**E**RYDQAENYGLVVGT	507
HsHACL2 (A1L0T0)	547	N**D**AGWTQISR**E**QVPSLGSNVAC	568
MmHACL2 (Q8BU33)	547	N**D**AGWTQISR**E**QVPRLGSDVAC	568
DrHACL2 (Q6NV04)	536	N**D**ACWSQISR**E**QVPMLGSNVAC	557
CeHACL (O61856)	554	N**D**ACWTQIAR**E**QVPMFQSSVAV	575

RuHACL (URHD0017)	467	N**N**GGIGPGMP**E**IP**E**NPMFNLKP	488
HsHACL1 (Q9UJ83)	481	N**N**NGIYQGF**D**T**D**TWK**E**MLKFQD	502
MmHACL1 (Q9QXE0)	484	N**N**NGIYQGF**D**A**D**TW**E**KMLHFQE	505

Figure S3. Structure-based alignment of WT AcHACL with other HACL and bacterial ALS enzymes. A 22-aa sequence segment including a conserved residue for Mg^{2+} coordination (N487 in AcHACL, highlighted in bold) and the 11-aa α-helix (yellow background) bearing a glutamic acid with presumably catalytic role (E493 in AcHACL, highlighted in red) is shown. Structures and AlphaFold models used are *E. coli* ALS, EcALS (PDB ID: 6LPI), BsALS (PDB ID: 4RJI), *Klebsiella pneumoniae* ALS, KpALS (PDB ID: 1OZF), AcHACL (PDB ID: 7PT1), HsHACL2 (AF-A1L0T0-F1), *Mus musculus* HACL2, MmHACL2 (AF-Q8BU33-F1), *Danio rerio* HACL2, DrHACL2 (AF-Q6NV04-F1) and *Caenorhabditis elegans* HACL, CeHACL (AF-O61856-F1). For comparison, the corresponding sequence segment is also shown for RuHACL (PDB ID: 6XN8) not possessing defined secondary structural features in the deposited structure. Additionally, the AlphaFold models for HsHACL1 (AF-Q9UJ83-F1) and *Mus musculus* HACL1, MmHACL1 (AF-Q9QXE0-F1) were considered, although the predicted 9-aa α-helix (grey background) is not homologous to the one found in AcHACL. For RuHACL and the two HACL1 sequences, candidate glutamic and aspartic acid residues possibly playing a related role to E493 in AcHACL are highlighted in blue. Accession numbers (in parentheses) refer to NCBI locus tag (for BsALS and AcHACL) or UniProt entry (all others).

Superposition of AcHACL with BsALS and AlphaFold model of HsHACL2: C-terminal lid region and adjacent α-helix bearing a presumably catalytic glutamic acid residue

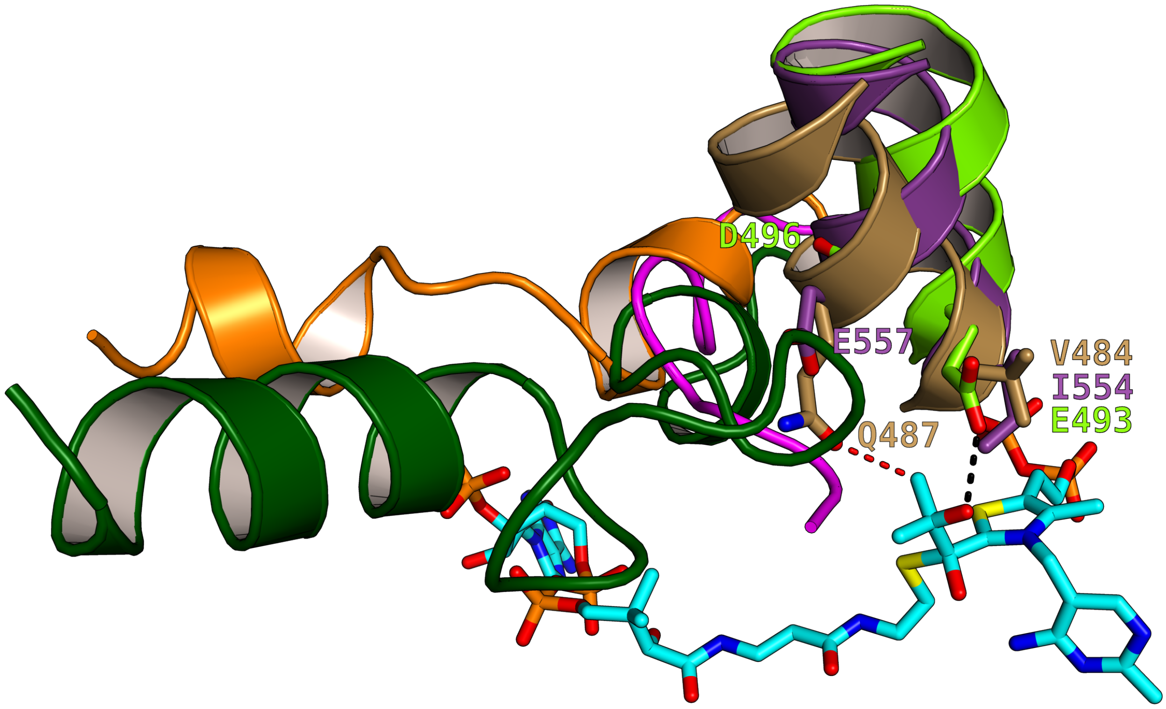

Figure S4. Superposition of the crystal structures of AcHACL (green) and BsALS (beige, PDB ID: 4RJI) with the AlphaFold model of HsHACL2 (violet, AF-A1L0T0-F1) shows that the active site α-helix is in a closed conformation for all three structures. The C-termini limit the closing angle of the α-helix, especially in AcHACL with its large lid domain and its helical breakout (dark green). In BsALS, the C-terminus (orange) is located away from the active site, thereby allowing a smaller closing angle. Residue E493 forms an interaction (dashed black line) with the 2-hydroxyl group of the modeled first intermediate (light blue) in AcHACL, whereas HsHACL2 and BsALS have an isoleucine and valine residue, respectively, at this position that prevent H-bonding interactions. However, in HsHACL2 and several related enzymes, a glutamic acid is present three residues later (one helix turn) in the sequence within the α-helix which is possibly involved in substrate interactions (see alignment in Figure S3). Due to the smaller closing angle in BsALS, the α-helix with glutamine residue Q487 would be at an appropriate distance to form an interaction with the AcHACL intermediate within this superposition (dashed red line), thereby illustrating a possible interaction mechanism for HsHACL2-related enzymes. The short C-terminus of HsHACL2 (magenta) modeled by AlphaFold is of low confidence, therefore, a smaller closing angle of the α-helix with residue E557 toward the intermediate appears possible in order to form an interaction between the side chain of E557 and the 2-hydroxyl group of the intermediate.

COBALT alignment of AcHACL with closest BLAST hits

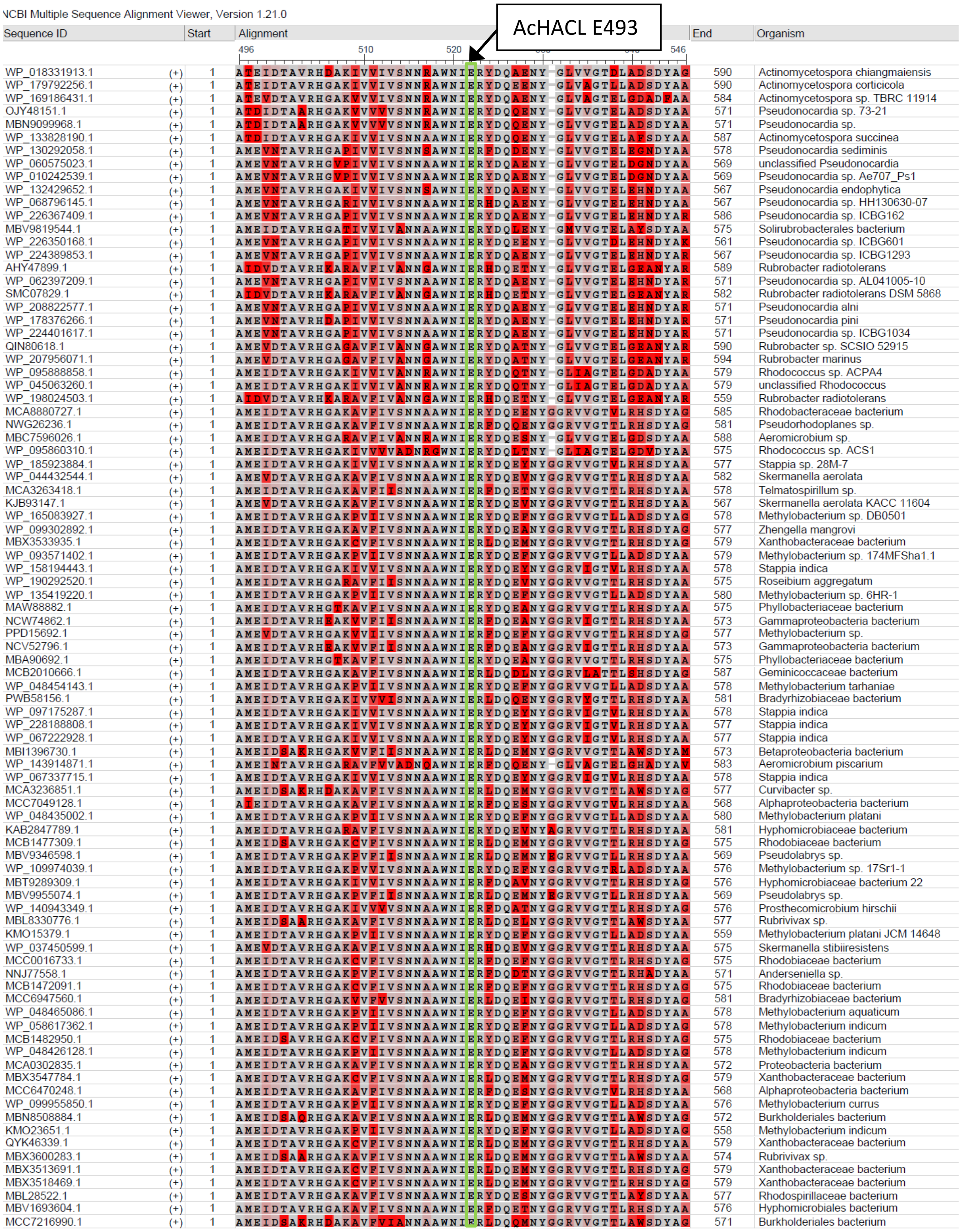

Figure S5. COBALT (Constraint-based Multiple Alignment Tool) alignment of closest BLASTP hits (≥59.8% sequence identity) with WT AcHACL sequence (NCBI locus tag WP_018331913.1) as query (against NCBI database of non-redundant protein sequences, November 2021). A 50-aa sequence segment including the conserved glutamic acid residue corresponding to E493 in AcHACL (highlighted with green box) is shown. Coloring code reflects frequency-based difference with darker shades of red indicate a further difference from residues in other rows in the alignment at that position.

Experimental details on enzyme assays and kinetic plots for WT AcHACL and mutants E493A and E493Q

Optimal pH and temperature for the WT AcHACL-catalyzed conversion of 2-HIB-CoA to acetone and formyl-CoA were determined as pH 7.2 and 37 °C, respectively, by incubating the enzyme at pH 5.9 to 8.6 (at 30 °C) and 30 to 45 °C (at pH 7.2) with 300 µM 2-HIB-CoA, 200 µM ThDP, 200 µM ADP and 5 mM $MgCl_2$ in 100 mM potassium phosphate buffer supplemented with 10% glycerol. Variation of ThDP concentrations within 80 to 1080 µM and ADP concentrations within 0 to 1000 µM had no effect on enzyme activity when tested at 30 °C and pH 7.2. With 100 mg L^{-1} of the E493K mutant enzyme (heterologous protein with 65,275.30 g mol^{-1}) incubated at 37 °C and pH 7.2 with 300 µM 2-HIB-CoA over 10 min, lyase activity was insignificant, i.e., it did not exceed the detection limit of ≤0.1 nmol min^{-1} mg^{-1} corresponding to the non-enzymatic decrease of 2-HIB-CoA observed under these assay conditions. Enzyme assays were performed discontinuously by taking samples at least three times over the incubation period and quantifying the residual 2-HIB-CoA concentration by HPLC. Rates of 2-HIB-CoA decrease were obtained by linear regression analysis. Depending on initial substrate concentration and enzyme activity, incubation time and amount of enzyme varied between 2.5 to 20 min and 10 to 100 mg L^{-1}, respectively.

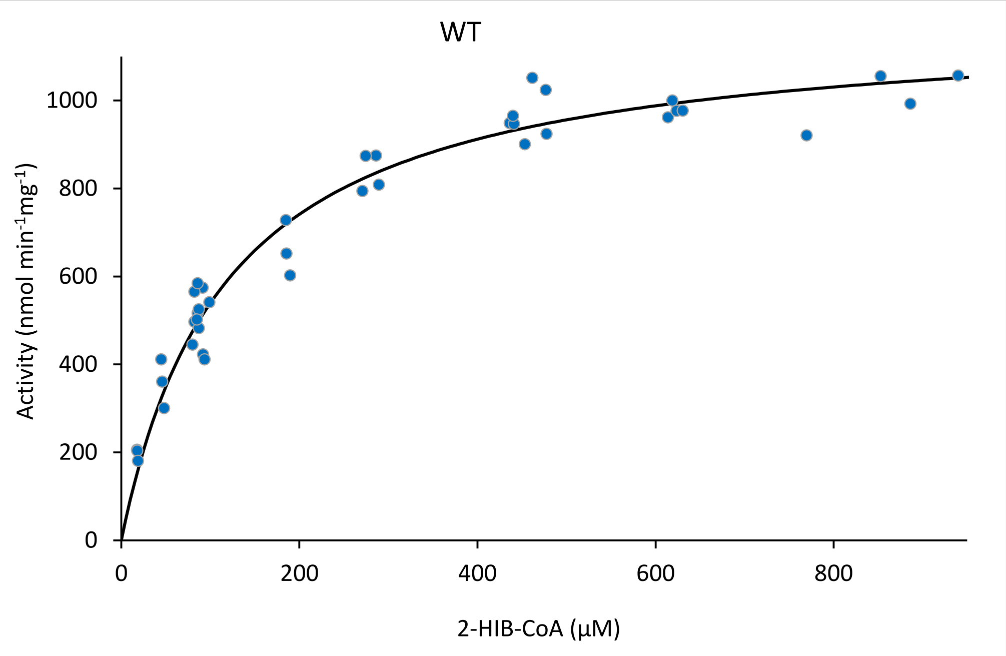

Figure S6. Kinetic plot for the conversion of 2-HIB-CoA to acetone and formyl-CoA catalyzed by WT AcHACL (heterologous protein with 65,276.24 g mol^{-1}) at pH 7.2 and 37 °C. Initial substrate concentrations ranged from 18 to 940 µM that were incubated with 10 or 20 mg L^{-1} enzyme. In total, 40 assays were performed. Nonlinear regression analysis gave the Michaelis-Menten fit as indicated with kinetic parameters ± SD as V_{max} = 1190 ± 34 nmol min^{-1} mg^{-1} (95% confidence interval 1108 to 1263 nmol min^{-1} mg^{-1}), K_m = 120 ± 12 µM (95% confidence interval 93.25 to 146.1 µM), R^2 = 0.991.

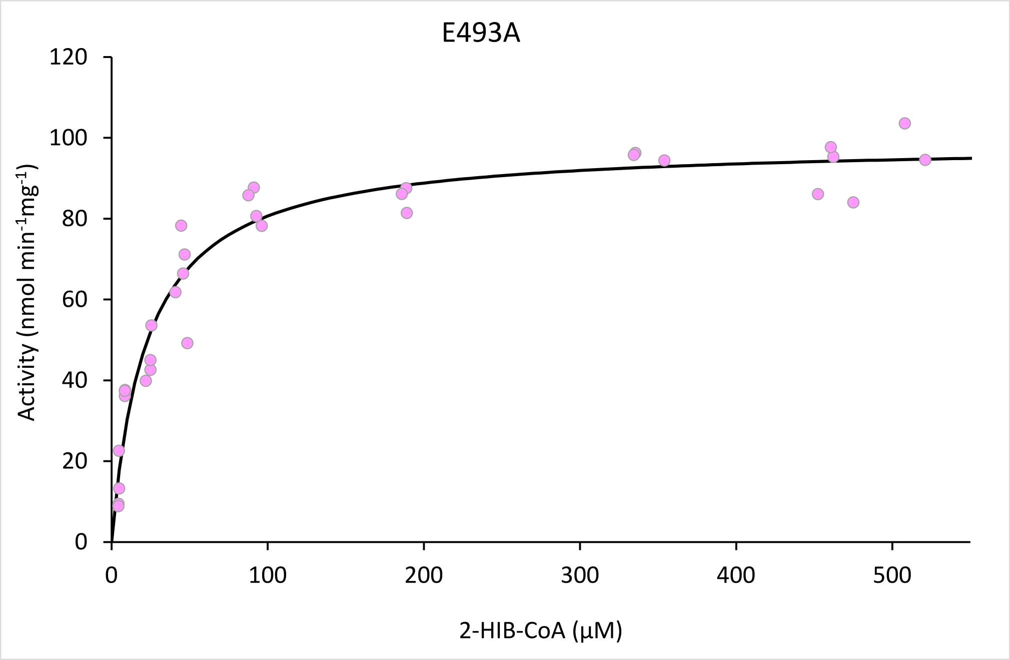

Figure S7. Kinetic plot for the conversion of 2-HIB-CoA to acetone and formyl-CoA catalyzed by AcHACL mutant E493A (heterologous protein with 65,218.21 g mol^{-1}) at pH 7.2 and 37 °C. Initial substrate concentrations ranged from 4.5 to 520 µM that were incubated with 20, 50 or 100 mg L^{-1} enzyme. In total, 32 assays were performed. Nonlinear regression analysis gave the Michaelis-Menten fit as indicated with kinetic parameters ± SD as V_{max} = 98.8 ± 3.5 nmol min^{-1} mg^{-1} (95% confidence interval 90.52 to 107.1 nmol min^{-1} mg^{-1}), K_m = 22.5 ± 3.5 µM (95% confidence interval 14.18 to 30.90 µM), R^2 = 0.983.

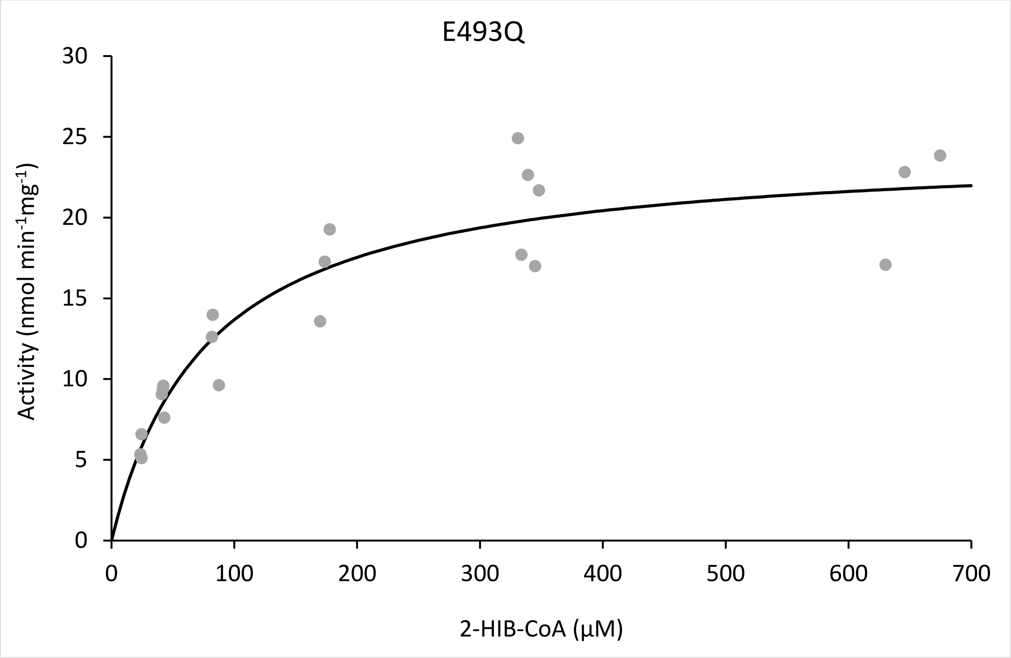

Figure S8. Kinetic plot for the conversion of 2-HIB-CoA to acetone and formyl-CoA catalyzed by AcHACL mutant E493Q (heterologous protein with 65,275.26 g mol^{-1}) at pH 7.2 and 37 °C. Initial substrate concentrations ranged from 24 to 675 µM that were incubated with 100 mg L^{-1} enzyme. In total, 21 assays were performed. Nonlinear regression analysis gave the Michaelis-Menten fit as indicated with kinetic parameters ± SD as V_{max} = 24.5 ± 0.7 nmol min^{-1} mg^{-1} (95% confidence interval 22.71 to 26.21 nmol min^{-1} mg^{-1}), K_m = 79.0 ± 7.1 µM (95% confidence interval 60.63 to 97.29 µM), R^2 = 0.996.
